# Trends and correlated outcomes in population-level prescription opioid and transdermal fentanyl use in Israel

**DOI:** 10.1186/s13584-023-00558-9

**Published:** 2023-03-20

**Authors:** Barak Shapira, Ronny Berkovitz, Ziona Haklai, Nehama Goldberger, Irena Lipshitz, Paola Rosca

**Affiliations:** 1 Division of Enforcement and Inspection, Ministry of Health, Jerusalem, Israel; 2 Health Information Division, Ministry of Health, Jerusalem, Israel; 3 Department for the Treatment of Substance Abuse, Ministry of Health, Jerusalem, Israel

**Keywords:** Prescription opioids, Fentanyl, Morbidity, Hospitalisations, Indicators, Population

## Abstract

**Background:**

In the last twenty years, there was a documented increase in prescription opioid procurement in Israel. However, there is still little evidence of the association between opioid procurement rates, health service utilisation in secondary care, and enrollment rates to substance use disorder treatment programmes. In this study, we show trends in the reports of opioid-related hospitalisations, emergency department visits, enrollment to community-based outpatient treatment for Prescription Opioid Use Disorder and opioid-related mortality rates. Additionally, we examine potential correlations between these health service utilisation rates and prescription opioid procurement rates at the population level, with a focus on transdermal fentanyl.

**Methods:**

A longitudinal study at the population level. We used seven-year data on indicators of opioid-related morbidity, prescription opioid procurement data for 2015–2021, and six-year opioid-related mortality data for 2015–2020. We measure the correlation between procurement rates of prescription opioids in Oral Morphine Equivalent per capita, and aggregated rates obtained from hospital administrative data for hospitalisations, emergency department visits, and patient enrolment in specialised prescription opioid use disorder outpatient treatment in the community setting.

**Results:**

Between 2015 and 2021, procurement rates in primary care per capita for all prescription opioids increased by 85%, while rates of transdermal fentanyl procurement increased by 162%. We found a significant positive correlation at the population level, between annual opioid procurement rates, and rates per population of opioid-related visits to emergency departments (r = 0.96, *p* value < 0.01, [CI 0.74–0.99]), as well as a positive correlation with the rates per population of patient enrolment in specialised prescription opioid use disorder outpatient treatment (r = 0.93, *p* value = 0.02, [CI 0.58–0.99]). Opioid-related mortality peaked in 2019 at 0.31 deaths per 100,000 but decreased to 0.20 deaths per 100,000 in 2020.

**Conclusion:**

Data shows that all-opioid and transdermal fentanyl procurement has increased yearly between 2015 and 2021. This increase is positively correlated with a growing demand for community-based Prescription Opioid Use Disorder outpatient treatment. Efforts to reduce opioid-related morbidity may require effective approaches toward appropriate prescribing, monitoring, and further increasing access to prescription opioid outpatient treatment.

## Background

Studies show associations between the overall rates of prescription opioid consumption at the population level, and indicators of opioid-related morbidity and mortality, including Emergency Department (ED) visits [1–3], hospitalisations [4], and fatal overdoses [5, 6]. The US opioid crisis, which was characterised in its initial stages by high levels of prescription opioid procurement, coupled with a rise in opioid-related overdoses, hospitalisations, and mortality is the most salient example of this association [7–9]. Similarly, an increase in prescription opioid use has also been observed across countries in mainland Europe such as France and Germany [10] and in the UK, albeit with a smaller overall impact on morbidity and mortality than that observed in the US [11]. These associations between levels of opioid use and opioid-related morbidity and mortality provide a strong rationale for reducing opioid exposure at the population level.

In the US, the COVID-19 pandemic coincided with a dramatic exacerbation in indicators of opioid-related mortality and morbidity, and an inflection point denoting a reversal of the gains made in previous years in reducing drug-related overdoses: The US Center for Diseases Control registered a 33% increase in fatal and non-fatal overdoses during the first year of the pandemic [12]. In addition, cardiac arrests related to drug overdose peaked at 50% above baseline pre-pandemic levels [13]. Not all countries were similarly affected during the pandemic, and rates of opioid-related hospitalisations and ED admissions varied considerably [14, 15]. For example, data from England and Wales in 2020 reveal only a small 5% increase in opioid-related deaths compared to pre-pandemic levels [16].

In Israel, data show that prescription opioid use has increased significantly in the past twenty years. Primary care data from the largest Health Maintenance Organisation (HMO) in Israel show a 68% increase in fulfillment of prescription opioids, measured in oral morphine milligram equivalent units (OME) between 2001 and 2004 [17], and a subsequent 7.3-fold increase in prescription opioid use among non-elderly patients with chronic pain [18]. The increase in prescription opioid utilisation is correlated with a growing incidence of opioid use disorder (OUD) related diagnoses such as drug dependence, drug abuse, overdose, and with rising healthcare expenditures [19]. It is important to contrast this data with previous ten-year data trends of opioid overdoses from Israel, suggesting an overall country-wide decrease in opioid-related deaths in the past decade [20, 21]. Nevertheless, the unequivocal data of the past twenty years, showing year-to-year increases in opioid use, has prompted the Israel Ministry of Health to consider new regulations and policies to mitigate what is now considered a public health problem. In 2018, the Director General of the Ministry of Health established an expert committee to advise on policies to reduce prescription opioid utilisation and misuse [22].

Additionally, the Israeli Association for Public Health and two of the state's HMOs have opened nine outpatient clinics to provide specialised treatment for patients diagnosed with Prescription Opioid Use Disorder (POUD). These programmes mostly operate evenings to separate POUD patients from those attending Opioid Maintenance Treatment (OMT) andreceiving racemic methadone or buprenorphine [23]. The POUD outpatient programmes provide pharmacological and psychosocial treatment to patients with non-severe OUD, not requiring hospitalisation [24].

In the United States, increases in illicit and licit fentanyl exposure have been highly correlated with, and explain most of the variation in overdose mortality in the past decade [25, 26]. Similarly, prescription transdermal fentanyl patches have been implicated in overdose deaths in Israel in the past two years in press reports, and by Ministry of Health officials [27, 28]. Moreover, the Israeli Ministry of Health has received reports by primary care physicians indicating a growing incidence of abuse of fentanyl transdermal formulations by patients with chronic pain, and use of transdermal fentanyl by people who use illicit drugs like heroin [29]. Procurement of fentanyl formulations in Israel has also increased by 200% between 2008 and 2018—a steeper and faster increase compared to other strong opioids such as oxycodone and buprenorphine [18].

In considering the need to provide up-to-date data on prescription opioid use in Israel, and the potential effects of the COVID-19 pandemic on opioid-related morbidity and mortality, this paper aims to describe pre-pandemic and pandemic prescription opioid utilisation trends. Moreover, it aims to describe correlations at the population level between prescription opioid procurement and indicators of opioid-related morbidity, mortality, and enrolment in POUD treatment. Finally, we provide year-by-year trends on transdermal fentanyl procurement using primary care data to provide further context of its contribution to the current increase of prescription opioid use in Israel.

## Methods

### Study design and data acquisition

This study uses a longitudinal, observational design to identify potential correlations between procurement rates of prescription opioids and indicators of opioid-related outcomes at the population level. The design looks at the Israeli population as the unit of analysis. We used seven-year data on indicators of opioid-related admittances at the population level, as reported by all Israeli public hospitals for the years 2015–2021. These hospital reports are based on administrative data processed from hospitals' automated systems. The data included primary and secondary diagnoses at discharge for hospitalisations and ED visits using the International Classification of Diseases, 9th edition (ICD-9) nomenclature. The codes checked were 96,500, 96,509, and E8502, which denote intentional or accidental opioid-related poisonings [30].

In addition, data on the number of patients treated for POUD at outpatient centres were obtained from annual reports provided to the Department for the Treatment of Substance Abuse of the Israel Ministry of Health. The aggregated number of hospitalisations, ED visits, and POUD patient enrolment numbers were converted to rates by dividing them by the mid-year Israeli population.

Lastly, we provide six-year aggregated mortality data from the Cause of Death database, maintained by the Israeli Central Bureau of Statistics and currently available until 2020. The Central Bureau of Statistics assigns the underlying cause of death, defined as the one leading to the fatality. The underlying cause is extracted from the notification forms filled by physicians at the time of death and is coded using the International Classification of Diseases 10^th^ edition (ICD-10). The deaths included in our analyses are those with an underlying cause of accidental, undetermined intent or deliberate poisoning by opioids (X42, Y12, X62) or other self-harm/suicide (X61, X63–64), assault/homicide (X85), poisoning of undetermined intent (Y11, Y13–Y14), mental health diagnoses associated with opioids (F11.0–F11.5, F11.7–F11.9), and opioid-related deaths, if they were coded together with one of the following ICD-10 codes: T40.0 (Opium), T40.1 (Heroin), T40.2 (Natural and semi synthetic opioids), T40.3 (Methadone), T40.4 (Synthetic Opioids, other than methadone), and T40.6 (Other unspecified narcotics) amongst contributing causes of death on the form.

Annual aggregated dispensing records for prescription opioids for the years 2018–2021 were obtained from publicly available data [31]. Data on opioid procurement for 2015–2017 was reported to the Division of Enforcement and Inspection by two HMOs providing healthcare coverage to approximately 66% of the Israeli population. The data includes all prescription opioids in Israel procured through the HMOs patient coverage scheme for patients not in the cancer register, but excludes formulations containing the weak opioids tramadol and codeine. The data also includes the following labels: formulation type, quantity, and brand name: (example: Tab oxycontin 80 mg/20 tab; Patch Fentanyl 25 mcg/hr/5 patches. Hence, the procurement rates shown pertain most closely to people with no cancer pain.

Dispensing data were converted using the OME methodology and standardised to the population covered by the HMOs. The OME methodology provides a way to compare opioids using a specified conversion factor so that different formulations are standardised to a single unit of measurement approximating their equianalgesic effects to those of oral morphine [32].

Since only two HMOs provided data for 2015–2017, the analysis was by population rates taking the yearly aggregated number of prescriptions for all opioids, converting them to OME, and dividing the total OME by the population number of people insured by the HMOs. Additionally, because data for the years 2015–2017 is partial, we used a backward interpolation linear model from the Python Pandas package (Python Software Foundation, Beaverton, OR) to estimate population OME rates for 2015–2017 based on rates from 2018 to 2021. Finally, the interpolated data and the calculated OME, based on HMO data, were compared. There was a 1.32% Mean Absolute Error indicating a good match between the predicted values, and those calculated using HMO data.

### Analysis

We carried out a separate analysis for all prescription opioids and fentanyl patches, as these were implicated in recent overdose cases reported to the Israeli Ministry of Health. We used Pearson’s correlation coefficient (r) and its associated 95% Confidence Intervals (95% CIs) and *p* values to measure the strength and direction of the relationship between two sets of variables: prescription opioid procurement rates in OME, transdermal fentanyl procurement rates in OME, and the per 100,000 population rates of indicators of opioid-related hospitalisations, ED visits, and POUD enrolment. Pearson’s correlation allows for examining the correlation between rates without assuming a normally distributed data. Python3 Sci-Py Library (Enthought, Austin, TX) was used for the analysis.

We provide visualisations of plots describing OME rates per capita for all opioids and transdermal fentanyl, hospitalisations and ED visits associated with prescription opioid use. We also provide a visualisation of trends in opioid-related death rates per 100,000 population. We finish by discussing current trends in prescription opioid use, the possible effect of the COVID-19 pandemic on opioid-related hospitalisations and ED visits, and the role of transdermal fentanyl in the current opioid public health problem in Israel.

## Results

### Indicators of opioid-related adverse outcomes

The population rates of POUD patient enrolment, opioid-related hospitalisations, and ED visits, related to opioid use varied during the seven years examined in our study. The rates of reported hospitalisations peaked in 2018 at 4.8 per 100,000 population and decreased to 2.0 per 100,000 in the period 2021. However, there was an overall decrease of 49% in opioid-related hospitalisation rates between 2015 and 2021.

**Fig. 1:**
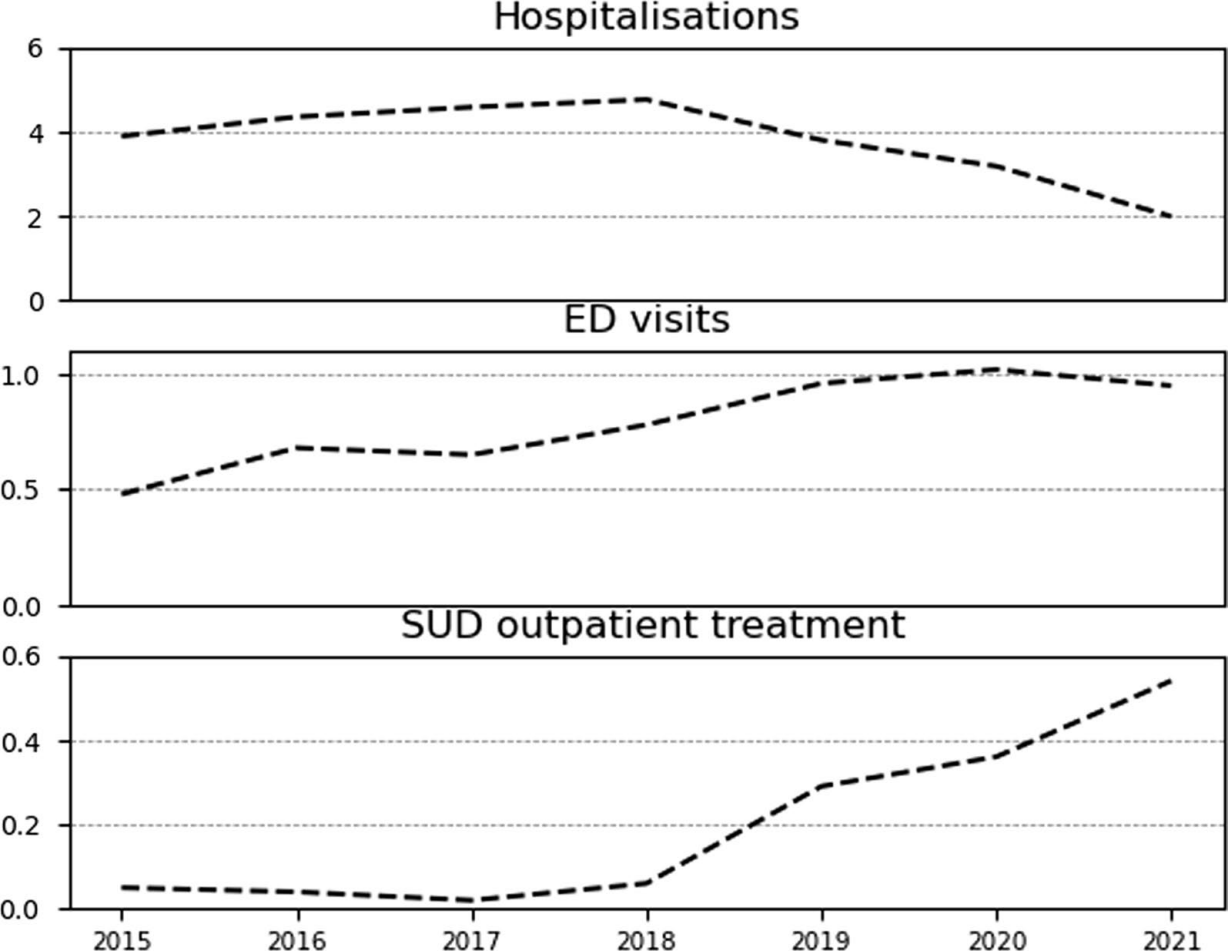
Annual trends of opioid-related outcomes per 100,000 population. *ED* Emergency department, SUD: Substance use disorder. Note: SUD outpatient treatment refers to specialised services for prescription opioid use

Until 2018, the number of patients enrolled in POUD programmes was negligible, not surpassing five individuals per year. However, from 2019, patient enrollment almost doubled, from 26 patients to 51 in 2021. Similarly, the rate of ED visits related to opioid use almost doubled between 2015 and 2021 from 0.48 per 100,000 population to 0.95. Annual trends of these opioid-related indicators are shown in Fig. 1, while the absolute number of cases reported for hospitalisations, ED visits, and reported opioid-related deaths are summarised in Table 1.

**Table 1:** Opioid related indicators by year in absolute numbers

Year	Patient enrollment in POUD treatment	Emergency department visits	Hospitalisations	Deaths
2015	4	40	326	17
2016	3	58	372	15
2017	2	57	399	11
2018	5	69	423	18
2019	26	87	344	28
2020	33	94	293	19
2021	51	90	188	–

### Opioid-related mortality

**Fig. 2:**
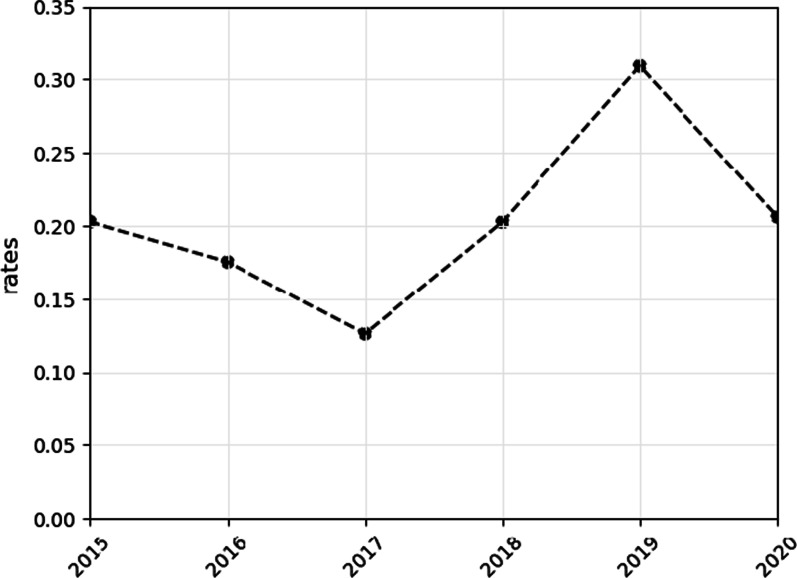
Opioid-related deaths per 100,000 population in Israel

Central Bureau of Statistics data show that between 2015 and 2020, the annual rates of opioid-related mortality varied considerably. Reported opioid deaths peaked in 2019 at 0.31 per 100,000, only to decrease again to 0.21 during the first year of the COVID-19 pandemic (Fig. 2). A comparison of overall rates reveals that despite the fluctuations, the was no change in the rates between years 2015 and 2020, both at 0.21 deaths per 100,000.

### Opioid utilisation rates

**Fig. 3:**
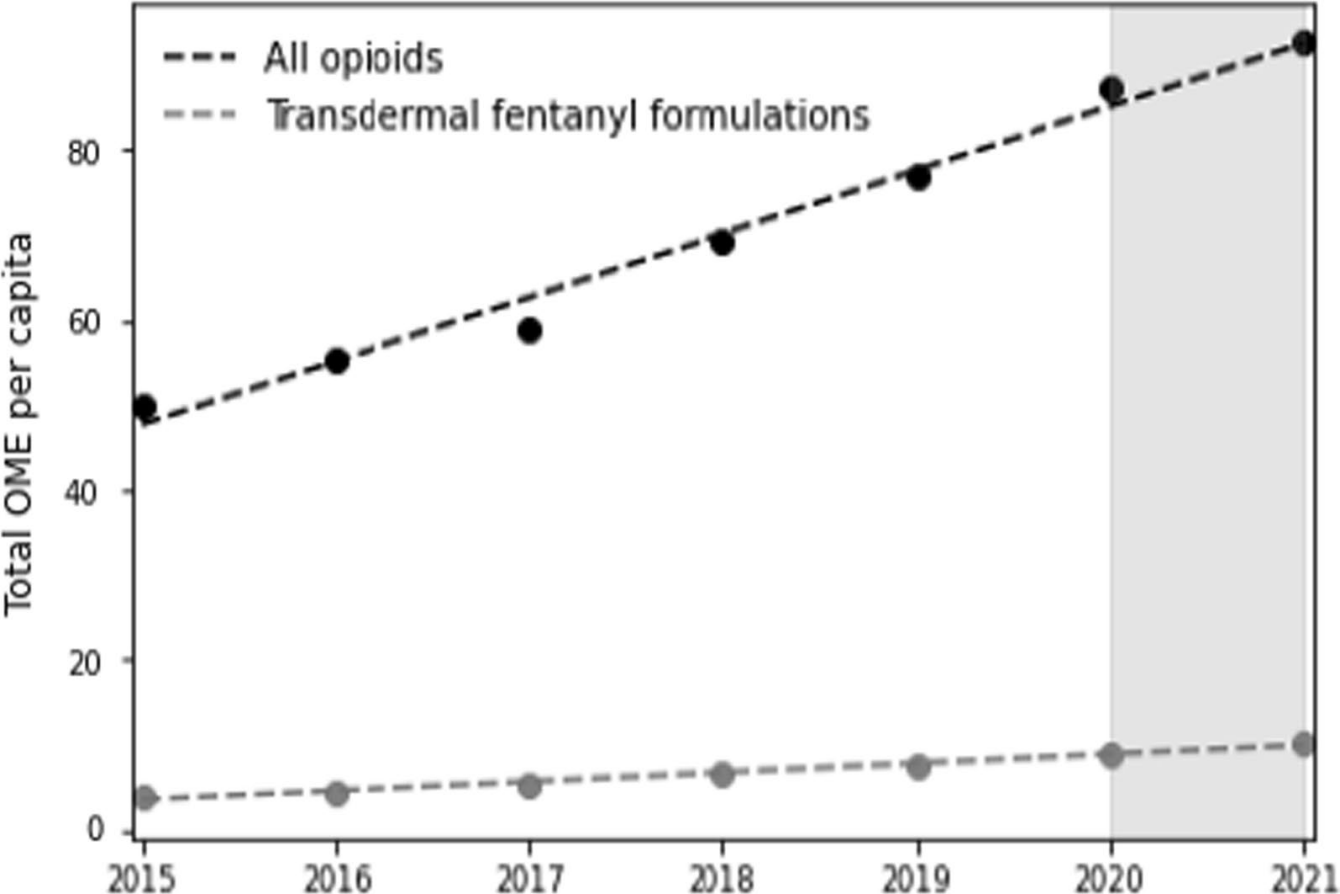
Per capita annual all-opioids, and transdermal fentanyl OME (in milligrams). *Note*: Vertical spans denote COVID-19 pandemic period. *OME*: Oral morphine equivalent

Data in Table 2 show a constant, year-by-year increase in all-opioid and transdermal fentanyl procurement for people with no-cancer pain. OME rates per capita for procurement of strong opioids increased by 85% between 2015 and 2021 to 92.6 mg per capita. Similarly, OME rates per capita of transdermal fentanyl patches increased by 162% between 2015 and 2021—now estimated to be 10.2 mg per capita. When comparing year-by-year percentage increases in Table 3, the dispensing of fentanyl patches grew at a faster pace than all-opioid dispensing rates per capita. The largest percent increase was recorded between 2017 and 2018 when strong opioid dispensing increased by 17.3% and transdermal fentanyl dispensing increased by 24%. Figure 3 provides a visualisation of the trends in the procurement of opioids and transdermal fentanyl. Data show only a slight deceleration in opioid procurement rates during the first two years of the COVID-19 pandemic, registering a smaller 6% increase.

**Table 2:** Estimated all-opioids, and TD fentanyl OME in grams

Year	All-opioid OME	TD fentanyl OME	TD fentanyl OME per capita (in mg)	All opioids OME per capita (in mg)
2015	419,690	19,603	3.9	50.1
2016	473,485	24,688	4.5	55.4
2017	513,942	30,469	5.4	59.0
2018	615,059	37,913	6.7	69.2
2019	695,591	43,888	7.6	76.8
2020	805,370	51,733	8.9	87.4
2021	874,540	60,116	10.2	92.6

*OME* Oral morphine equivalent, *TD* Transdermal

**Table 3:** Percent change in OME per capita, compared to previous year

Year	Transdermal fentanyl	All opioids
2015	–	–
2016	15.4	10.6
2017	20.0	6.5
2018	24.1	17.3
2019	13.4	11.0
2020	17.1	13.8
2021	14.6	5.9

*OME* Oral morphine equivalent

### Correlation between opioid OME rates and indicators of opioid-related adverse outcomes

There is a significant positive correlation at the population level between annual OME rates and opioid-related visits to the ED per 100,000 population (r = 0.96, *p* value < 0.01, [CI 0.74–0.99]). There is also a significant positive correlation between annual OME rates and patient enrolment for POUD outpatient treatment per 100,000 population (r = 0.93, *p* value < 0.01, [CI 0.58–0.99]).

However, no significant positive correlations were found between all opioid OME rates and hospitalisations. Moreover, no significant positive correlations were found between OME per population data and mortality data for years 2015–2020. Due to similar trends in transdermal fentanyl use, positive correlations were also shown for transdermal fentanyl and ED visits (r = 0.94, *p* value < 0.01, [CI 0.63–0.99]), and transdermal fentanyl and POUD patient enrolment (r = 0.93, *p* value < 0.01, [CI 0.58–0.99]).

## Discussion

The present research provided seven-year data on prescription opioid procurement. It also aimed to identify correlations between annual procurement levels of opioids and transdermal fentanyl, indicators of opioid-related admittances in secondary care, and POUD outpatient enrolment. This research also included data from the first two years encompassing the COVID-19 pandemic.

Results demonstrate, as in previous studies from other countries, that there is a significant positive correlation between opioid procurement at the population level and a growing incidence of some opioid-related health services utilisation indicators [10, 11, 33, 34]. This positive association was established for diagnoses related to opioid use, employing primary care data in Israel [18]. However, here we provide further evidence for such an association with some, but not all, events in the hospital setting, such as ED visits, and enrolment to specialised POUD treatment services.

An important finding was that hospitalisation trends were not positively correlated with opioid procurement trends. Here, we show that reports of hospitalisations have been decreasing since 2017. In comparison, opioid-related ED visits per population increased dramatically since 2017 and were highest in 2020, slightly decreasing in 2021. The discrepancy between the increase in ED visits and decreased hospitalisations associated with opioid use should be further explored. A possible explanation for these differing trends in hospitalisation and ED visits stems from the possible convergence of two processes. On the one hand, there has been an increase in the past five years in outpatient OMT and Substance Use Disorder (SUD) inpatient and residential ambulatory programmes, offering patients with both POUD and OUD more options for treatment at the community setting [24]. On the other hand, there is an ongoing problem in providing a continuum of care for patients who left or were discharged from inpatient or residential programmes—most diagnosed with an OUD and using illicit opioids such as heroin. A recent report by the Knesset Research and Information Centre claims that in 2017, more than 40% of patients did not finish a full treatment term in inpatient services, and only 30% were further referred to social services and treatment communities [35]. While we cannot measure the effect of this deficiency in the provision of a continuum of care on all people with OUD, we can assume that it leaves many patients without proper treatment, further exacerbating their condition. This situation could partly explain why many more unstable patients with OUD end up in the ED rather than in community care or hospitalisation.

Opioid-related mortality rates continued to fluctuate considerably during the period of analysis. The lack of data from 2021 makes it extremely difficult to discern if opioid-related deaths are rising or descending again. Following the increase in deaths between 2018 and 2019, we showed a subsequent significant decrease in death rates in 2020. Considering 10-year rates between 2011 and 2020, the overall trend remains constant [20, 21]. It is also possible that the "Notification of Death" form does not always document opioid use among the causes of death. We conclude that current mortality data do not provide evidence of an opioid overdose crisis in Israel. However, the negative outcomes of opioid use may not be reflected only in mortality data and should be assessed by their impact on the well-being and morbidity of patients. This situation reveals a different aspect of the Israel opioid problem, which differs from that of the US and is not reflected in rising overdose deaths. For comparison, opioid-related deaths in the US, calculated similarly, reveal rates above 30 per 100,000 population, 100 times larger than those shown in our study [12]. Similarly, albeit calculated differently, opioid-related death rates per 100,000 population in England and Wales reveal rates ten times larger than those reported in Israel [16]. Possible reasons for the comparatively low rates observed in Israel have been discussed in a previous review [21]. These reasons include information bias due to misclassification of the underlying causes of death related to opioids, a universal health system that mitigates severe OUD cases, and different drug-market circumstances that currently do not involve the large-scale distribution of potent illicit fentanyl analogues.

Except for the changing trends in mortality during 2020, which are consistent with the periodic fluctuations observed in previous years, there were no dramatic changes in the rates of hospitalisations, ED visits, POUD enrollment, and opioid procurement during the first year of the COVID-19 pandemic. The results shown in this study should be contrasted with the experiences of other countries, where patients faced difficulty accessing specialised outpatient SUD treatment. During the COVID-19 pandemic, many people with SUD reported problems accessing treatment due to lockdowns and reduced service hours, and some renounced going or could not access hospital emergency care [36]. In Israel, during the early stages of the pandemic, SUD treatment services faced closures, limited services, and experienced staff shortages due to mandatory isolations and restrictions [37]. Nevertheless, in contrast to the experience of other countries, it appears that in Israel, the COVID-19 pandemic cannot be associated with a dramatic change in trends of reported opioid-related events in secondary care, as these have established themselves years before the pandemic. At the same time, and further contributing to the decrease in hospitalisation trends seen in data from previous years—more people with SUD were possibly discharged or not admitted for hospitalisation to allow staff to deal with the COVID-19 emergency.

Incidentally, results show a growing enrolment for specialised POUD treatment, with patients' rates doubling during the pandemic years. The growing rates in POUD treatment enrolment should be viewed as the outcome of growing demand and increased availability, as more programmes have opened during the past five years. From 2014 to 2018, only three centres provided specialised treatment for POUD in Central and Northern Israel. In the three years between 2019 and 2021, three more centres were added to the fold, two from the north and one from the Jerusalem area. In 2022, three more programmes opened—one serving the south of Israel and two serving the Israeli Arab population.

Our results also show a worrying trend pointing to an increase in opioid procurement by Israeli HMOs in primary care, now estimated to be at over 90 OME per capita rates. It should be noted that our findings only estimate procurement in primary care for  strong opioids among patients not in the cancer registry. Thus, actual procurement figures in OME per capita could be thrice as much, or four times  those estimated here if country-wide figures for all indications from the secondary, tertiary, army and private health sectors are counted. Such a hypothetical procurement figure of 740 OME per 1000 population per day would place Israel over some estimations for 2019 Western European consumption (estimated between 250 and 480 OME per 1000 population per day) and in the range of those of the UK (estimated at 639 OME to 1500 OME per 1000 population per day) and the US (estimated at 738 OME to 1000 OME per 1000 population per day) [38, 39].

Another trend of concern relates to fentanyl consumption in Israel. Here we documented growing rates of transdermal fentanyl use in primary care. In the context of country-wide consumption, Israel, with its population of 9.3 million, is among the top 10 consumers of fentanyl, with 43.9 kg or 3.8% of the annual reported world consumption. These numbers are higher than those of the UK (2.5%), the Netherlands (3.2%), and Canada (2.7%) [40]. Upon closer examination, transdermal fentanyl dispensing increases annually faster than opioid dispensing. This faster growth in fentanyl dispensing suggests that the substance may have a prominent role in the current opioid problem in Israel.

A full analysis of prescribing policies and systemic factors that allowed for the rise in prescription opioid use in Israel is beyond the scope of this article. However, a rudimentary examination of some salient factors is possible. One contributing factor stems from the clinical practices prevalent among clinicians in the first decade of the twenty-first century, which advocated an aggressive approach towards pain management and a less restrictive attitude towards opioid prescribing than in previous decades [41]. Increased patient awareness and demand for strong opioids may have also contributed to this increase [42]. In this context, transdermal fentanyl doses of 75 mcg and above are extremely potent and effective analgesics, delivering doses above 180 OME per day and are very easy to use by patients through skin application.

In addition, local prescribing policies may have contributed to the current situation: First, although prescribing laws in Israel place nominal limits on the daily doses of some opioids, physicians can prescribe doses above those limits in cases of chronic or severe disease. Moreover, in contrast to opioids like oxycodone, morphine, or pethidine, the law does not limit the daily dose of fentanyl medication that may be dispensed by a pharmacist [43]. In addition, there is no centralised database collecting dispensing data from all Israeli pharmacies. The lack of a centralised database makes identification of dispensing from multiple pharmacies to a single patient difficult and may facilitate misuse use of prescription opioids. Lastly, we should not preclude the contribution of the illicit drug market to the increased demand for strong prescription opioids. A study among people with SUD enrolled in treatment in Israel points to the growing use of fentanyl and strong opioids as a substitute for heroin [44]. Thus, prescription fentanyl is being diverted to non-medical users, possibly playing a small role in the rise of total consumption.

### Policy recommendations

The opioid use problem in Israel is multifactorial and addressing it may require a synthesis of measures. We recommend addressing the opioid problem through improvement of four main areas. First, policies should support the rationalisation of opioid prescribing among clinicians. Current clinical guidelines on opioid use for pain emphasise that the risks of long-acting formulations like transdermal fentanyl in patients with chronic non-cancer pain outweigh their benefits and recommend using non-opioid alternatives in chronic pain [45]. Second, clinicians should be trained on up-to-date standards of care for patients experiencing chronic pain, and training should be readily accessible and actively provided by HMOs and hospitals. In this regard, the Israel Ministry of Health has requested that all public health services, such as hospitals and HMOs, institute a prescription opioid rationalisation scheme to decrease opioid use in non-cancer chronic pain by pre-authorising strong opioids like oxycodone and fentanyl in non-cancer patients. However, under no circumstances should access to these medicines be curtailed for patients requiring treatment.

Third, follow-up of opioid-prescribed patients should become a priority for Health Care Providers. Clinicians should routinely administer risk assessment questionnaires to patients, such as the Screener and Opioid Assessment for Patients with Pain-Revised (SOAPP-R) and the Opioid Risk Tool (ORT) to identify patients at risk and those that may have developed OUD. Primary care clinicians should also be trained in opioid tapering and refer patients to SUD treatment if needed. The Israel Ministry of Health is currently providing qualification courses for prescribers on using buprenorphine for chronic pain and SUD treatment courses. These need to be expanded and disseminated among primary care prescribers so that they gain more confidence and competence in prescribing and tapering opioids.

The Israel Medical Association has made an important step in promoting good prescribing practices through its 2016 position paper on opioid treatment for non-cancer pain [46]. However, these guidelines need to be updated and strengthened, particularly by conveying a clear message regarding the preference for short-acting opioids for chronic pain, and relaying the complexities of using transdermal fentanyl in non-cancer chronic pain conditions.

Fourth, interventions must be implemented to curtail the inappropriate prescribing of opioids and prevent opioid misuse. For example, new regulations on fentanyl could be placed to limit dose and availability as initial treatment in non-cancer pain. Moreover, up-to-date pharmacy dispensing data should be available by linking pharmacies to HMOs. This linkage will help to identify patients acquiring opioids from multiple healthcare providers and pharmacies. Additionally, new regulations should allow reporting of opioid procurement rates annually to the Ministry of Health [47]. As of September 2022, the Ministry of Health is proposing amendments to the Israel drug ordinance and its regulations to limit the dose and length of treatment with opioids used in non-palliative care [30].

Lastly, authorities should strive to improve access to treatment, given the rising enrollment rates for specialised POUD services. Improving access is crucially important when tightening restrictions on opioid prescribing. Tightening the “regulatory ratchet” too strongly without providing treatment alternatives to those already suffering from OUD could paradoxically lead to more opioid-related deaths. In addition, harsh limits on access to prescription opioids may encourage users to find alternative means of obtaining them or turn to illegal opioids from the street drug market [48].

Future research should help discern cases of prescription opioid use from those of illicit opioids such as heroin. For example, primary care and pharmacy dispensing studies could help compare health outcomes among patients using opioids in high doses (> 90 OME per day). Additionally, research with linked pharmacy dispensing data could help identify patients with aberrant behaviours, such as those acquiring prescription opioids in high doses, and from multiple prescribers ("doctor shopping") or different pharmacies. Identifying patients engaging in opioid misuse could help healthcare providers target patients for much needed interventions.

## Limitations

### Use of administrative data to infer opioid-related outcomes

The non-uniform reporting patterns in administrative data among clinicians and data coders may lead to misclassifications in reporting opioid-related events. As a result, using ICD-9 and ICD-10 codes often leads to underestimating the real incidence of opioid-related events. For example, validation studies show that inference of opioid overdose events from ICD-9 and ICD-10 codes had low sensitivity and relatively high specificity [49, 50]. Similarly, a study made among a sample of people who use drugs in Melbourne revealed that ICD-10 codes could only reasonably infer 42% of opioid-related outcomes queried [51]. Thus, caution should be exercised when interpreting the results of this study.

### Other limitations

It is important to note that the associations between opioid dispensing and our indicators on a population level do not necessarily indicate causality. Accordingly, using just seven points corresponding to the years of analysis for the Pearson correlation results in very large confidence intervals and potentially missing other meaningful associations. Additionally, data on drug utilisation and dispensing in Israel is mired by incomplete year-to-year data, requiring estimations and imputation techniques. The data indicates procurement by HMOs only for non-cancer patients, while actual country-wide procurement, which includes private consumption and procurement in hospitals, may be significantly larger than that reported in this study. Finally, our data do not include cases found in hospitals and medical centres specialising in mental health, nor does it include data on OMT utilisation trends which could have provided another robust outcome, greatly strengthening our conclusions.

## Conclusion

The study shows that opioid utilisation has increased significantly between 2015 and 2021 in Israel. With this increase in opioid use, the demand for and delivery of specialised treatment for POUD has also increased. Furthermore, data also show that opioid utilisation has continued to increase during the COVID-19 pandemic and that transdermal fentanyl is a prominent contributor to this increase. The latest mortality data reveals a low, yet highly fluctuating rate throughout the past decade, contrasting the Israeli opioid problem with that of the US, Canada or the UK. Given the dynamic situation and rising procurement rates, collecting up-to-date information on opioid-related outcome measures, instituting policies on rationalising prescription opioids, improving patient follow-up, and improving access to POUD treatment should become a priority for health care providers.

## Data Availability

The aggregated datasets supporting the conclusions of this article are made available by the corresponding author upon reasonable request.

## Abbreviations

ED: Emergency department
ICD: International Classification of Diseases
HMO: Health Maintenance Organisation
OME: Oral morphine milligram equivalent
OMT: Opioid maintenance therapy
OUD: Opioid use disorder
POUD: Prescription opioid use disorder
SUD: Substance use disorder
